# Co‐pathologies modify hippocampal protein accumulation patterns in neurodegenerative diseases

**DOI:** 10.1002/alz.14355

**Published:** 2024-12-23

**Authors:** Koji Yoshida, Shelley L. Forrest, Shojiro Ichimata, Hidetomo Tanaka, Tomoya Kon, Gabor G. Kovacs

**Affiliations:** ^1^ Department of Laboratory Medicine and Pathobiology and Department of Medicine University of Toronto Toronto Ontario Canada; ^2^ Tanz Centre for Research in Neurodegenerative Disease Krembil Discovery Tower University of Toronto Toronto Ontario Canada; ^3^ Department of Legal Medicine Graduate School of Medicine and Pharmaceutical Sciences University of Toyama Toyama Japan; ^4^ Laboratory Medicine Program & Krembil Brain Institute University Health Network Toronto Ontario Canada; ^5^ Department of Neurology Dementia Research Centre Macquarie Medical School Faculty of Medicine Health and Human Sciences Macquarie University Sydney Australia; ^6^ Graduate School of Medicine Hirosaki University Hirosaki Japan

**Keywords:** alpha‐synuclein, Alzheimer's disease, amyloid beta, corticobasal degeneration, hippocampus, limbic predominant age‐related TDP‐43 encephalopathy neuropathological change, Lewy body disease, phosphorylated tau, progressive supranuclear palsy, TDP‐43

## Abstract

**INTRODUCTION:**

Limited research has extensively analyzed neurodegenerative disease–related protein deposition patterns in the hippocampus.

**METHODS:**

This study examined the distribution of proteins in hippocampal subregions across major neurodegenerative diseases and explored their relation to each other. The area density of phosphorylated tau (p‐tau), amyloid beta (Aβ), α‐synuclein, and phosphorylated TDP‐43 protein deposits together with pyramidal cell density in each hippocampal subregion, including CA1‐4, prosubiculum (ProS), and subiculum was assessed in 166 cases encompassing various neurodegenerative diseases.

**RESULTS:**

Alzheimer's disease‐associated p‐tau predominated in ProS, Aβ in the CA1, and Lewy body–related α‐synuclein in the CA2. The area density of protein deposits increased with the pathological stage until a peak, then decreased in cases with high pathology stages along with pyramidal cell density. Comorbid protein pathology influenced protein deposition patterns.

**DISCUSSION:**

This comprehensive evaluation reveals characteristic neurodegenerative disease–related protein accumulation patterns in hippocampal subregions modified by co‐pathologies.

**Highlights:**

- Alzheimer's disease–related phosphorylated tau predominates in the prosubiculum.
- Amyloid beta predominates in the CA1 and Lewy body–related α‐synuclein in the CA2.
- The area density of protein deposition increases with the disease stage up to a peak.
- In the high pathology stage, protein deposition and pyramidal cell density decreases.
- Comorbid protein pathology affects the pattern of protein accumulation.

## 1 BACKGROUND

The hippocampus, a vital brain region linked to memory,^1^, ^2^ is often impacted by numerous neurodegenerative diseases.^3^ Comprising multiple subregions, including CA1–4 and the subiculum (Sub), each subregion serves distinct functions and exhibits different vulnerabilities depending on the underlying neurodegenerative disease.

The most extensively studied subregion is CA1, the predilection site for Alzheimer's disease (AD) and TDP‐43 proteinopathies.^4^, ^5^, ^6^, ^7^, ^8^, ^9^, ^10^ CA2 has also garnered attention, with notable accumulation of abnormal proteins in Lewy body disease (LBD),^11^ prion diseases,^12^ chronic traumatic encephalopathy (CTE),^13^ and primary age‐related tauopathy (PART),^14^ along with four‐repeat tauopathies like progressive supranuclear palsy (PSP), corticobasal degeneration (CBD), and argyrophilic grain disease (AGD).^15^ However, which subregion predominates in multiple system atrophy (MSA) remains unclear.

Furthermore, there has been limited investigation into the involvement of the prosubiculum (ProS), a subregion strongly linked to the amygdala located between CA1 and Sub,^16^ in neurodegenerative diseases. A previous report suggested that this region may be affected earlier than CA1 in AD,^17^ necessitating separate evaluation in diseases other than AD.

Exploring whether mixed pathologies influence the distribution patterns of each aberrant protein represents an understudied aspect of neurodegenerative disease pathology. Neurodegenerative diseases often coexist,^18^, ^19^ particularly in the elderly, and biochemical interactions between abnormal proteins have been reported. In addition to the association between tau protein and amyloid beta (Aβ) protein in AD postulated in the frame of the amyloid cascade hypothesis,^20^ those involving Aβ, tau protein, and alpha‐synuclein have also been reported.^21^, ^22^, ^23^, ^24^, ^25^, ^26^ However, limited research has investigated whether further proteins influence the distribution patterns of a specific protein in the brain. On the other hand, the distribution of abnormal proteins is a crucial indicator of disease progression, exemplified by the Braak neurofibrillary tangle (NFT) stage of tau protein in AD.^27^ Hence, it is imperative to reconsider whether using these indices for a specific protein is preferable in cases affected by copathologies,^28^ or distinct progression patterns can be recognized.

Based on these aspects, our objective in this study is to comprehensively determine the distribution of each aberrant protein in the hippocampal subregions, including the ProS, of major neurodegenerative diseases and further explore whether the presence of further proteins alters their distribution patterns.

## 2 METHODS

### 2.1 Case selection

A retrospective review of 166 neuropathological autopsies from the University Health Network Neurodegenerative Brain Collection (UHN‐NBC) was conducted. All brains have been obtained at autopsy through appropriate consenting procedures with local ethics committee approval by the UHN Research Ethics Board (Nr. 20‐5258) and the University of Toronto (Nr. 39459) and was performed per the ethical standards established in the 1964 Declaration of Helsinki, updated in 2008.

**RESEARCH IN CONTEXT**

1. **Systematic review**: While it is known that neurodegenerative diseases often coexist, particularly in elderly individuals, the impact of mixed pathologies on the distribution patterns of each abnormal protein has not been thoroughly explored.
2. **Interpretation**: Based on the comprehensive evaluation of the hippocampal subregions, we demonstrated that neurodegenerative comorbidities influence the distribution patterns of abnormal protein accumulation in each other. These findings align with previously less extensively reported results.
3. **Future directions**: In the future, it would be desirable to comprehensively demonstrate how neurodegenerative comorbidities influence each other in regions beyond the hippocampus, similar to our findings. Such an endeavor has the potential to reshape the current approach to neurodegenerative disease pathology, in which the distribution of abnormal proteins typically gauges severity. This paradigm shift may also extend to imaging assessments of the brain.

AD‐related pathology was evaluated based on the Braak NFT stage,^27^ Thal phase,^29^ and CERAD (Consortium to Establish a Registry for Alzheimer's Disease) criteria.^30^ The Alzheimer's disease neuropathologic change (ADNC) level was determined using the ABC score as defined by the National Institute on Aging—Alzheimer's Association (NIA‐AA) guidelines.^31^ The severity of Lewy‐related pathology was assessed following the Lewy disease consensus criteria^32^ using α‐synuclein immunohistochemistry. Cases with intermediate and high ADNC levels were classified as AD, those with severe Lewy‐related pathology of the limbic or neocortical type as LBD, and those meeting both criteria as AD+LBD. PSP^33^ and CBD^34^ were diagnosed according to established criteria, AGD was classified based on the Saito stage,^35^ TDP‐43 pathology was categorized according to limbic predominant age‐related TDP‐43 encephalopathy neuropathological changes (LATE‐NC) stage.^36^ These stages were diagnosed through comprehensive screening of the neocortex and white matter (including the frontal, parietal, occipital, and temporal lobes), basal ganglia, amygdala, ambient gyrus, cingulate gyrus, hippocampus, entorhinal cortex, midbrain, pons, medulla oblongata, and cerebellum, as thoroughly as possible. The diagnoses were conducted by KY, SLF, SI, HT, and TK and confirmed by GGK.

A total of 166 cases were included in this cohort, comprising 31 AD, 38 AD+LBD, 16 LBD, 36 PSP, 5 CBD, 14 MSA, and 26 controls. Controls were defined as cases with No or Low ADNC levels and no other major degenerative disease (Table 1). Cases in the control group were primarily used to determine cutoff thresholds for pyramidal cell loss and for regression analysis alongside cases in the other disease groups.

**TABLE 1:** Case summary.

	Control	AD	AD+LBD	LBD	MSA	PSP	CBD	
	(*n *= 26)	(*n *= 31)	(*n *= 38)	(*n *= 16)	(*n *= 14)	(*n *= 36)	(*n *= 5)	*P value*
**Age, mean ± SD, years**	65.8 ± 14.3	77.9 ± 10.3	75.3 ± 9.4	76.7 ± 8.5	66.3 ± 8.1	75.7 ± 7.1	67.0 ± 6.4	<0.001
**Sex (male/female)**	17/9	9/18	17/18	11/4	6/6	19/17	3/1	0.37
**ADNC level, mean ± SD**	0.6 ± 0.5	2.9 ± 0.3	2.5 ± 0.5	0.8 ± 0.4	0.4 ± 0.5	0.9 ± 0.8	0.8 ± 0.8	<0.0001
** A Score, mean ± SD**	0.8 ± 0.8	3.0 ± 0.0	2.6 ± 0.5	1.2 ± 0.9	0.6 ± 0.9	1.1 ± 1.1	1.0 ± 1.0	<0.0001
** B Score, mean ± SD**	0.7 ± 0.5	2.9 ± 0.3	2.7 ± 0.5	1.2 ± 0.4	0.9 ± 0.5	1.4 ± 0.6	1.0 ± 0.3	<0.0001
** C Score, mean ± SD**	0.7 ± 0.9	2.9 ± 0.3	2.6 ± 0.6	0.9 ± 0.8	0.3 ± 0.6	0.9 ± 1.0	0.8 ± 0.8	<0.0001
**Lewy related pathology positive, *n* (%)**	0 (0.0)	13 (43.3)	38 (100.0)	16 (100.0)	0 (0.0)	11 (30.6)	2 (40.0)	<0.0001
**LATE‐NC positive, *n* (%)**	0 (0.0)	9 (29.0)	20 (52.6)	3 (18.8)	1 (7.1)	6 (16.7)	1 (20.0)	<0.0001
**AGD positive, *n* (%)**	1 (4.0)	5 (16.1)	6 (15.8)	8 (50.0)	3 (21.4)	17 (47.2)	2 (40.0)	<0.001
**Vessel Score, mean ± SD**	2.7 ± 1.7	3.7 ± 1.4	3.7 ± 1.5	4.0 ± 1.2	4.3 ± 1.3	4.7 ± 1.0	3.4 ± 1.6	<0.0001
***APOE* gene genotype**								
** ε2/ε2, *n* (%)**	0 (0.0)	0 (0.0)	0 (0.0)	0 (0.0)	0 (0.0)	0 (0.0)	0 (0.0)	<0.01
** ε2/ε3, *n* (%)**	0 (0.0)	0 (0.0)	1 (3.2)	0 (0.0)	0 (0.0)	1 (4.2)	1 (33.3)	
** ε2/ε4, *n* (%)**	0 (0.0)	0 (0.0)	0 (0.0)	1 (8.3)	1 (7.7)	3 (12.5)	0 (0.0)	
** ε3/ε3, *n* (%)**	1 (100.0)	6 (85.7)	6 (19.4)	8 (66.7)	9 (69.2)	13 (54.2)	2 (66.7)	
** ε3/ε4, *n* (%)**	0 (0.0)	1 (14.3)	20 (64.5)	3 (25.0)	3 (23.1)	6 (25.0)	0 (0.0)	
** ε4/ε4, *n* (%)**	0 (0.0)	0 (0.0)	4 (12.9)	0 (0.0)	0 (0.0)	1 (4.2)	0 (0.0)	
***MAPT* gene haplotype**								
** H1/H1, *n* (%)**	1 (100.0)	5 (71.4)	15 (48.4)	9 (75.0)	11 (84.6)	22 (91.7)	3 (100.0)	0.51
** H1/H2, *n* (%)**	0 (0.0)	1 (14.3)	14 (45.2)	2 (16.7)	1 (7.7)	2 (8.3)	0 (0.0)	
** H2/H2, *n* (%)**	0 (0.0)	1 (14.3)	2 (6.5)	1 (8.3)	1 (7.7)	0 (0.0)	0 (0.0)	

**Abbreviations**: AD, Alzheimer's disease; ADNC, Alzheimer's disease neuropathological change; AGD, argyrophilic grain disease; *APOE*, apolipoprotein E; CBD, corticobasal degeneration; LATE‐NC, limbic‐predominant age‐related TDP‐43 encephalopathy neuropathologic change; LBD, Lewy body disease; MSA, multiple system atrophy; PSP, progressive supranuclear palsy; SD, standard deviation.

### 2.2 Pathological evaluation

Formalin‐fixed, 4.5‐µm paraffin‐embedded tissue sections of the posterior hippocampus from all 166 cases underwent staining with hematoxylin, eosin, and Luxol fast blue (HE/LFB), along with immunohistochemistry for phosphorylated tau (p‐tau, clone AT8, 1:1000, Invitrogen/ThermoFisher), Aβ (clone 6F/3D, 1:50, Dako), α‐synuclein (clone 5G4, 1:4000, Analytikjena),^37^ and phosphorylated TDP‐43 (pTDP‐43, clone 11‐9, 1:2000, CosmoBio). For certain p‐tau–positive cases, additional staining for 3‐repeat‐tau (3R‐tau, clone 8B6/C11, RD3, 1:5000, MilliporeSigma) and 4‐repeat‐tau (4R‐tau, clone 1E1/A6, RD4, 1:200, MilliporeSigma) was conducted. All immunostaining procedures were performed using Dako Autostainer Link 48 and EnVision FLEX+ Visualization System following the manufacturer's instructions.

**FIGURE 1:**
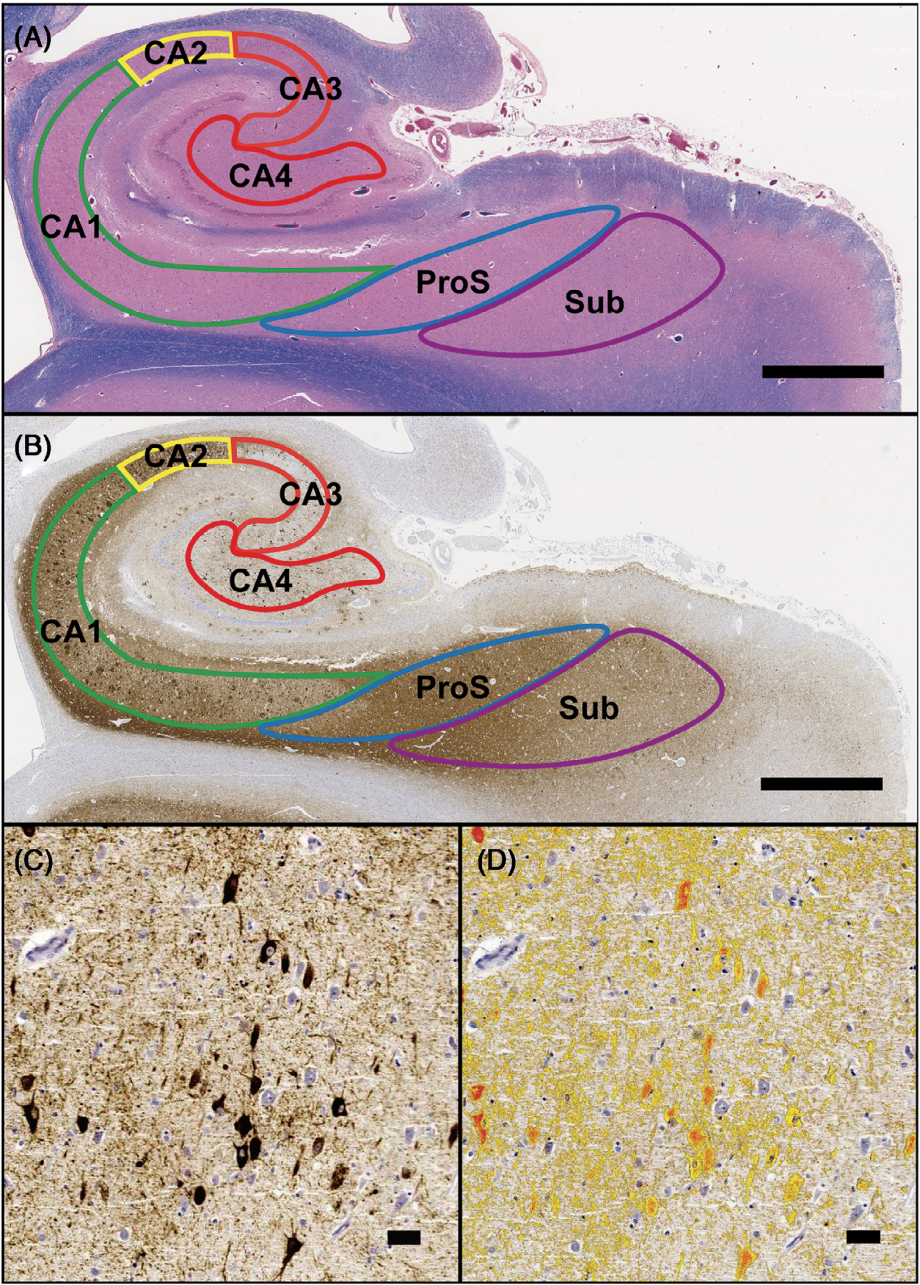
Subregion annotation and positive area measurement method. We annotated each subregion of CA1–4, prosubiculum (ProS), and subiculum (Sub) with an image of the posterior hippocampal section scanned with hematoxylin, eosin, and Luxol fast blue (A), and subsequently, each immunostained scan image was annotated to delineate that region (B: AT8). The positive area for each protein in each subregion was then assessed; (C) depicts the image before evaluation, while (D) shows the positive area in the same image highlighted in red. Scale bar: 1  mm (A‐B), 100  µm (C‐D).

Subregions of the posterior hippocampus, including CA1–4, ProS, and Sub pyramidal cell layers, were delineated following established anatomical criteria.^16^, ^38^, ^39^ KY conducted the annotations, which were reviewed by TK and GGK (Figure 1).

The posterior level (lateral geniculate body) of the hippocampus was immunostained for p‐tau, Aβ, α‐synuclein, and pTDP‐43 were digitalized at 40x magnification using a TissueScope LE120 (Huron Digital Pathology) and stored as comprehensive whole‐slide images. Subsequently, the images underwent analysis using the Area Quantification module of HALO software (Indica Labs). The area density of positive immunostaining in each subregion (3984 regions) was quantified for all 166 cases (664 sections).

Pyramidal cell density in each subregion was determined by capturing photographs at three locations in CA1 and one location each in CA2–4, ProS, and Sub, using a 100x field of view for each case. Imaging positions were randomly chosen within each subregion while avoiding small ischemic lesions (Figure S1A in supporting information). Pyramidal cells with visible nuclei in each image were manually counted using ImageJ and HALO software. For CA1, the mean density of the three locations was calculated. If the pyramidal cell layer of CA2 and CA3 was smaller than the 100x field of view, the density was calculated only within the field of view, excluding areas outside the pyramidal cell layer. To verify the accuracy of this pyramidal cell count estimation, a total of 72 subregions in Nissl‐stained posterior hippocampal sections from 12 randomly selected cases were counted using HALO software. Spearman correlation coefficient analysis showed a strong correlation (ρ = 0.8611, *P* < 0.0001), confirming the accuracy of the manually counted estimates (Figure S2 in supporting information). The pyramidal cell loss was defined as a cutoff of –2.5 standard deviation from the pyramidal cell density observed in the control group for each subregion (Figure S1B‐D). Hippocampal sclerosis (HS) cases were defined as those with pyramidal cell loss and reactive astrocytes in the CA1 region.

The Vessel Score, a modification of the Vascular Cognitive Impairment Neuropathology Guidelines (VCING),^40^ was used to measure cerebrovascular disease (CVD), and blood vessels were assessed in the frontal lobe and basal ganglia^41^ (Figure S3 in supporting information).

### 2.3 Genetic analysis

Apolipoprotein E (*APOE*) and microtubule‐associated protein tau (*MAPT*) genotypes were examined in 88 of the total 166 cases, as previously described.^42^ The genotypes for each group are listed in Table 1.

### 2.4 Statistical analysis

Chi‐square and Kruskal–Wallis tests were used to evaluate the variability among the groups. The Mann–Whitney *U* test was used to compare immunostaining positivity density and subregional comparison scores between the two groups, while the Steel–Dwass test was used for three or more groups. Regression analysis (analysis of covariance [ANCOVA]) was also conducted to evaluate the background pathology affecting these scores. In this regression analysis, the high Braak NFT stage was defined as ≥ 3, the high Thal phase as ≥ 3, the severe Lewy pathotype as limbic or neocortical, the high AGD stage as Saito stage ≥ 2, and the high LATE‐NC stage as ≥ 2. These analyses were performed using SPSS statistics version 26 (IBM Corporation), JMP 14.3 (JMP Statistical Discovery LLC), and GraphPad Prism version 9.5.1 (GraphPad Software). The significance level was set at *P *< 0.05.

## 3 RESULTS

### 3.1 Protein accumulation patterns

**FIGURE 2:**
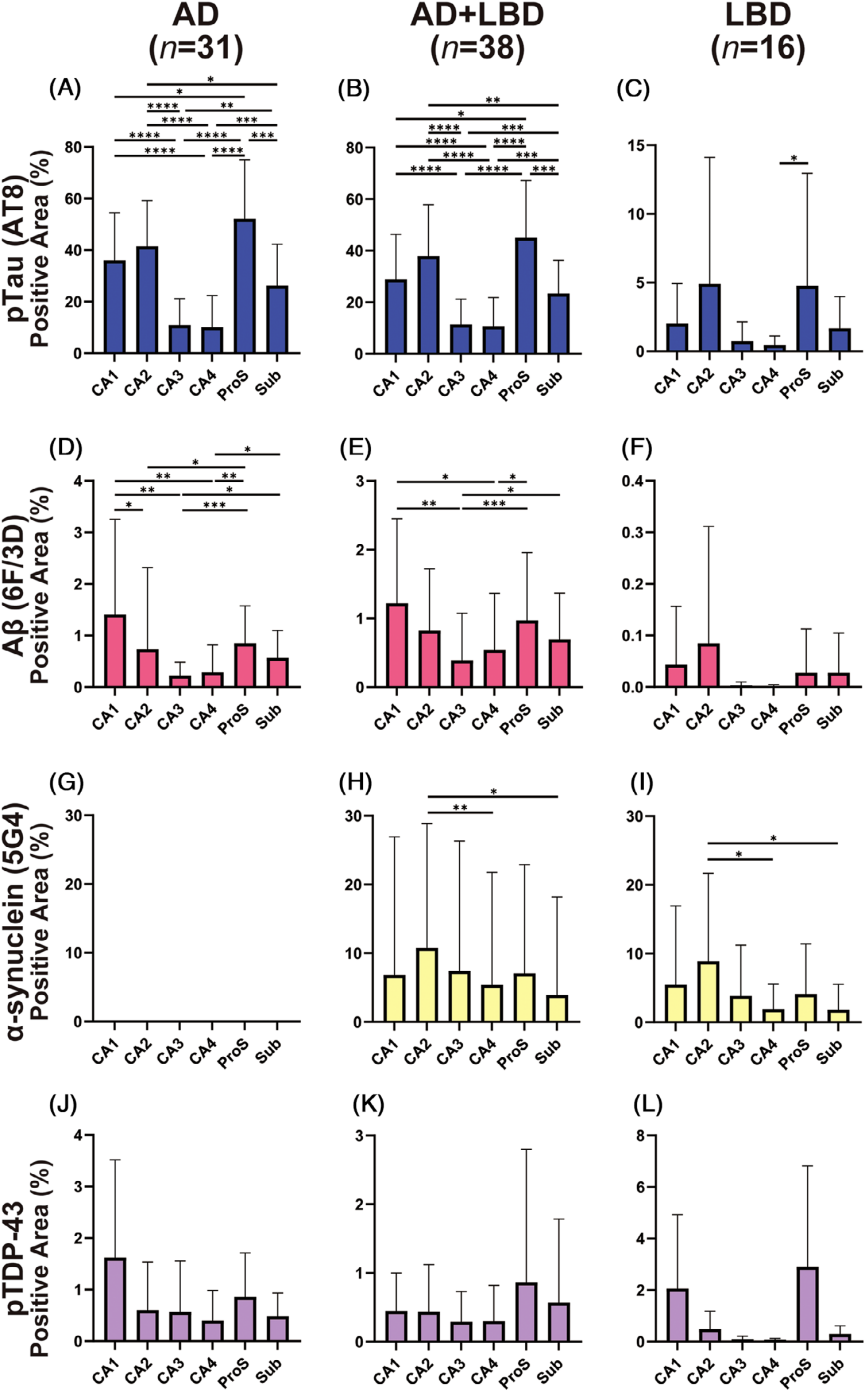
Protein accumulation in each subregion of AD, AD+LBD, and LBD groups. AD (A, D, G, J), AD+LBD (B, E, H, K), and LBD (C, F, I, L) were assessed for accumulation of p‐tau (AT8, A‐C), Aβ (6F/3D, D‐F), α‐synuclein (5G4, G‐I), and pTDP‐43 (J‐L) in each subregion of the hippocampus. pTDP‐43 pathology was detected in 9/31 AD, 20/38 AD+LBD and 3/16 LBD cases (see Table 1). Statistical significance was determined using the Steel–Dwass test (*P < 0.05, **P < 0.01, ***P < 0.001, ****P < 0.0001). Aβ, amyloid beta; AD, Alzheimer's disease; LBD, Lewy body dementia; p‐tau, phosphorylated tau; pTDP‐43, phosphorylated TAR DNA‐binding protein 43.

The mean area density of p‐tau deposition was most severe in the ProS, followed by the CA2, CA1, Sub, CA3, and CA4 in all groups except the LBD, PSP, and control groups. In the LBD group, the mean p‐tau accumulation density of CA2 was higher than that of ProS. In the PSP group, CA2 had a lower mean p‐tau accumulation density than CA1 and Sub, but the order of the other subregions was consistent (Figure 2A‐C, Figure S4A‐D in supporting information). In PSP cases without comorbid AGD, the mean p‐tau accumulation in CA2 was slightly higher than in CA1, but the predominance of ProS remained (Figure S5A, B in supporting information).

The involvement of the mean area density of Aβ deposition followed the order of involvement of CA1, ProS, CA2, Sub, CA4, and CA3 from highest to lowest in the AD and AD+LBD groups, while CA2 was highest in the LBD group, although it accumulated less than in AD (Figure 2D‐F). The mean area density of α‐synuclein deposition was highest in CA2 in the AD+LBD, LBD, and MSA groups, but the second and subsequent accumulation levels differed in each group (Figure 2G‐I, Figure S4I‐L). The mean area density of pTDP‐43 deposition was highest in the AD group for CA1 followed by the ProS. The ProS had the highest mean area density of protein deposition in the AD+LBD and LBD groups (Figure 2J‐L, Figure S4J‐L). This pattern varied depending on the presence or absence of pyramidal cell loss. In cases without pyramidal cell loss, a higher average area density of protein deposition was detected in the CA1 and ProS regions. However, in cases with pyramidal cell loss, there was no predominance of these subregions, indicating that the loss of pyramidal cells may influence the distribution of protein deposition across the hippocampal subregions (Figure S5C, D).

### 3.2 Comparison of each protein accumulation using a comparison score

The findings suggested that the predominance of accumulated proteins in CA1, CA2, and ProS could vary across the AD, AD+LBD, and LBD groups. Therefore, we specifically examined these three regions to validate this observation. To facilitate the comparison, we introduced a scoring system to assess protein accumulation between two regions, as outlined in the following equation:

Region Comparison Score = (Density of Protein Accumulation in Region A – Density of Protein Accumulation in Region B) / (Density of Protein Accumulation in Region A + Density of Protein Accumulation in Region B).

Cases in which no accumulation was observed in the compared regions were assigned a score of 0.

**FIGURE 3:**
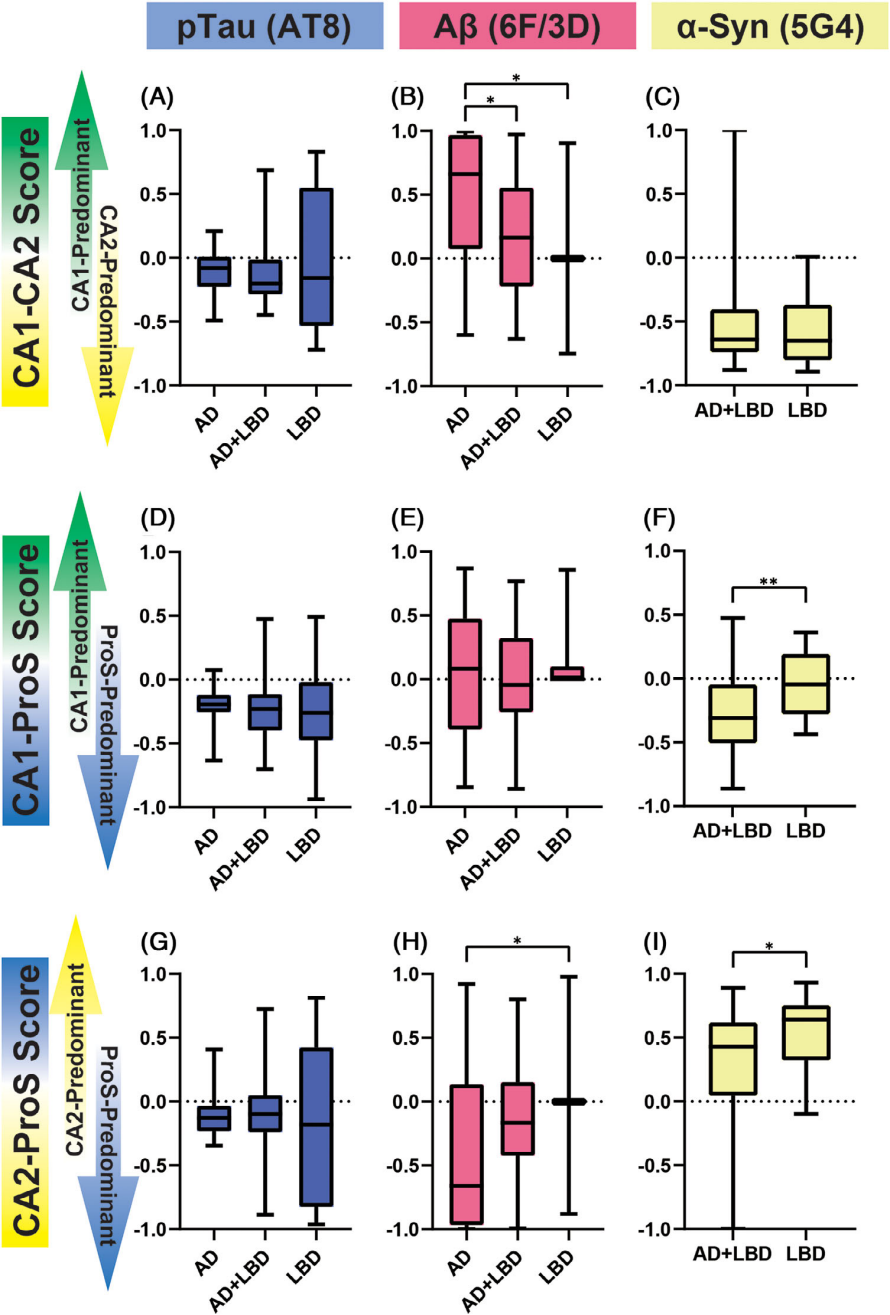
Comparison score for each protein in each group. The p‐tau scores for CA1–2 (A‐C), CA1‐ProS (D‐F), and CA2‐ProS (G‐I), as well as the Aβ scores (B, E, H) and α‐synuclein scores (C, F, I), were compared among the three groups: AD, AD+LBD, and LBD. While no significant differences were observed in the p‐tau scores between the groups, the CA1–2 score for Aβ was significantly lower in LBD (indicating CA2 predominance), and the CA2‐ProS score was significantly higher in LBD (also indicating CA2 predominance) compared to AD. Additionally, the CA1‐ProS score for α‐synuclein was significantly higher in the LBD group compared to the AD+LBD group (indicating CA1 predominance), and the CA2‐ProS score was significantly higher in the LBD group (indicating CA2 predominance). Statistical significance was determined using the Steel–Dwass and Mann–Whitney *U* tests (*P < 0.05, **P < 0.01). Aβ, amyloid beta; AD, Alzheimer's disease; LBD, Lewy body dementia; ProS, prosubiculum; p‐tau, phosphorylated tau.

The findings revealed that the AD group exhibited a notably higher CA1‐CA2 Comparison Score for Aβ compared to the AD+LBD and LBD groups. Additionally, the AD group displayed a significantly lower CA2‐ProS Comparison Score for Aβ compared to the LBD group. Furthermore, the AD+LBD group demonstrated significantly lower CA1‐ProS Score and CA2‐ProS Comparison Score for α‐synuclein than the LBD group. However, no significant differences were observed in p‐tau Comparison Scores among the groups (Figure 3).

### 3.3 Regression analysis for protein comparison scores

To comprehensively assess the interaction of protein distribution while accounting for the coexistence of AGD and LATE‐NC and the impact of neuronal loss, regression analysis (ANCOVA) was used to compare the Braak NFT stage, Thal phase, AGD Saito stage, LATE‐NC stage, and the two compared pyramidal cell densities of each of the two regions being compared as explanatory variables. This analysis was performed across the control, AD, AD+LBD, and LBD groups, excluding the PSP and CBD groups, to reduce the influence of tau pathology other than that associated with AD and to assess the impact of each background pathology on the Comparison Scores.

Pyramidal cell density was included to account for the possibility that a decrease in pyramidal cell density would result in less accumulation of each protein. Still, this analysis confirmed the opposite of what was expected, namely protein accumulation increased with decreasing pyramidal cell density (Table S1 in supporting information). From this result, we inferred that the relationship between pyramidal cell density and the amount of each type of accumulated protein was not linear. Thus, we determined that it was not suitable for regression analysis. Subsequently, we performed the analysis again, excluding pyramidal cell density from the explanatory variables.

The results indicated that a higher Braak NFT stage was significantly associated with a decreased CA1‐2 Comparison Score of p‐tau, a higher Thal phase with increased CA1‐2 Comparison Scores of p‐tau and Aβ, and with decreased CA1‐ProS and CA2‐ProS Comparison Scores of p‐tau. Limbic or neocortical type Lewy pathology was significantly associated with decreased CA1‐CA2 Comparison Scores for Aβ and α‐synuclein, CA1‐ProS Comparison Scores for α‐synuclein, and increased CA2‐ProS Comparison Scores for α‐synuclein. A higher AGD Saito stage was significantly associated with a decreased CA1‐ProS Comparison Score of p‐tau. A higher LATE‐NC stage was significantly associated with increased CA1‐CA2 Comparison Score of pTDP‐43, the CA1‐ProS Comparison Score of p‐tau, the CA2‐ProS Comparison Scores of p‐tau and α‐synuclein and was significantly associated with decreased CA1‐ProS and CA2‐ProS Comparison Scores of pTDP‐43 (Table 2).

**TABLE 2:** Regression analysis (ANCOVA) for protein comparison scores.

	CA1‐CA2 score															
	p‐tau (AT8)				Aβ (6F/3D)				α‐Synuclein (5G4)				pTDP‐43			
Explanatory variable (target side)	Estimated value	95% CI		*P* value	Estimated value	95% CI		*P* value	Estimated value	95% CI		*P* value	Estimated value	95% CI		*P* value
**Braak NFT stage** (high)	**−0.131**	**−0.204**	**−0.003**	**0.0178**	0.026	−0.114	0.166	0.7159	−0.001	−0.075	0.072	0.9748	0.026	−0.053	0.106	0.5164
**Thal phase** (high)	**0.125**	**−0.239**	**−0.023**	**0.0240**	**0.145**	**0.004**	**0.286**	**0.0435**	0.033	−0.041	0.107	0.3793	−0.041	−0.121	0.039	0.3176
**Lewy pathology type** (severe)	−0.011	0.017	0.233	0.7411	**−0.103**	**−0.189**	**−0.017**	**0.0188**	**−0.274**	**−0.319**	**−0.229**	**−0.0001**	−0.023	−0.072	0.026	0.3591
**AGD Saito stage** (high)	0.014	−0.078	0.056	0.7573	−0.007	−0.117	0.103	0.8948	−0.009	−0.067	0.049	0.7501	0.004	−0.059	0.067	0.9014
**LATE‐NC stage** (high)	−0.049	−0.074	0.101	0.2489	0.047	−0.062	0.156	0.3960	−0.042	−0.099	0.016	0.1525	**0.118**	**0.054**	**0.181**	**0.0004**
	**CA1‐ProS score**															
	**p‐tau (AT8)**				**Aβ (6F/3D)**				**α‐Synuclein (5G4)**				**pTDP‐43**			
	Estimated value	% CI		*P* value	Estimated value	95% CI		*P* value	Estimated value	95% CI		*P* value	Estimated value	95% CI		*P* value
**Braak NFT stage** (high)	−0.018	−0.105	0.069	0.6765	−0.061	−0.187	0.066	0.3427	−0.040	−0.111	0.031	0.2671	−0.020	−0.082	0.042	0.5205
**Thal phase** (high)	**−0.099**	**−0.186**	**−0.012**	**0.0266**	0.076	−0.050	0.203	0.2348	−0.015	−0.087	0.057	0.6778	0.031	−0.031	0.094	0.3219
**Lewy pathology type** (severe)	−0.047	−0.101	0.006	0.0815	−0.001	−0.079	0.076	0.9762	**−0.100**	**−0.144**	**−0.057**	**−0.0001**	−0.014	−0.052	0.025	0.4798
**AGD Saito stage** (high)	**−0.087**	**−0.157**	**−0.016**	**0.0162**	−0.090	−0.190	0.009	0.0735	0.051	−0.005	0.107	0.0764	−0.014	−0.063	0.035	0.5626
**LATE‐NC stage** (high)	**0.085**	**0.017**	**0.152**	**0.0145**	0.028	−0.070	0.126	0.5719	0.050	−0.005	0.106	0.0746	**−0.057**	**−0.106**	**−0.008**	**0.0239**
	**CA2‐ProS score**															
	**p‐tau (AT8)**				**Aβ (6F/3D)**				**α‐Synuclein (5G4)**				**pTDP‐43**			
	Estimated value	95% CI		*P* value	Estimated value	95% CI		*P* value	Estimated value	95% CI		*P* value	Estimated value	95% CI		*P* value
**Braak NFT stage** (high)	0.088	−0.038	0.215	0.1699	−0.056	−0.219	0.106	0.4938	−0.050	−0.142	0.041	0.2771	−0.025	−0.106	0.056	0.5432
**Thal phase** (high)	**−0.173**	**−0.301**	**−0.046**	**0.0081**	−0.072	−0.235	0.091	0.3848	−0.033	−0.125	0.058	0.4735	0.044	−0.038	0.126	0.2897
**Lewy pathology type** (severe)	−0.016	−0.094	0.063	0.6885	0.098	−0.002	0.197	0.0544	**0.186**	**0.130**	**0.241**	**<0.0001**	0.008	−0.042	0.059	0.7394
**AGD Saito stage** (high)	−0.074	−0.177	0.029	0.1562	−0.054	−0.182	0.073	0.3999	0.053	−0.019	0.125	0.1465	0.001	−0.063	0.065	0.9706
**LATE‐NC stage** (high)	**0.116**	**0.017**	**0.215**	**0.0224**	−0.089	−0.216	0.037	0.1624	**0.094**	**0.023**	**0.165**	**0.0101**	**−0.143**	**−0.208**	**−0.079**	**<0.0001**

**Abbreviations**: Aβ, amyloid beta; AGD, argyrophilic grain disease; ANCOVA, analysis of covariance; CI, confidence interval; LATE‐NC, limbic dominant age‐related TDP‐43 encephalopathy neuropathological changes; NFT, neurofibrillary tangle; p‐tau, phosphorated tau; pTDP‐43, phosphorylated TAR DNA‐binding protein 43.

*Notes*: Statistically significant findings are highlighted in bold, and subregions influenced by the target side are color‐coded as follows: CA1 in green, CA2 in yellow, and Prosubiculum in blue, which corresponds to Figures 1, 3, and Figure S1 in supporting information. The coloring of the pathological stages in the explanatory variable corresponds to Figures 2, 3, 4, 6, and Figure S2 in supporting information.

Finally, because significant variations in genetic background, age, and CVD pathology were observed in each disease group (Table 1), we performed sensitivity analyses by adding age and vessel score (Table S2 in supporting information) *APOE* ε4 and *MAPT* H1/H1 genotypes (Table S3 in supporting information) as explanatory variables. The results indicated that these variables did not significantly impact the association between protein comparison scores and background pathological stages. However, age and sex significantly altered the p‐tau CA1‐2 comparison score, while genotype did not result in any significant changes.

### 3.4 Protein accumulation and neuronal loss with disease progression

**FIGURE 4:**
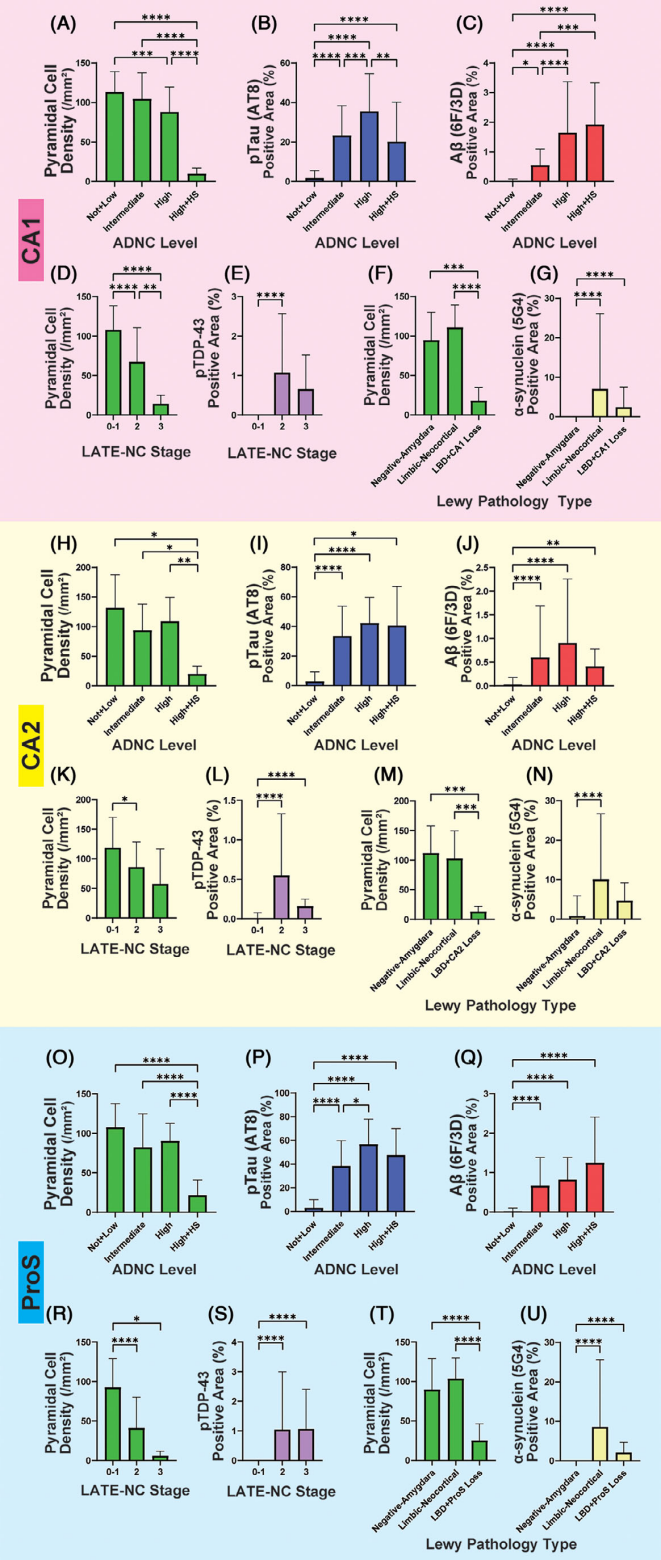
Relationship between pyramidal cell density and accumulated protein density in disease progression. In CA1 (A‐G), CA2 (H‐N), and Prosubiculum (ProS, O‐U), we evaluated the protein‐positive density (B‐C, E, G) for each ADNC level (A‐C, H‐J, O‐Q), LATE‐NC stage (D‐E, K‐L, R‐S), and Lewy disease type (F‐G, M‐N, T‐U), respectively, as well as the pyramidal cell density (A, D, F, H, K, M, O, R, T). Cases with neuronal loss in each subregion were separately assessed as HS (hippocampal sclerosis) and CA2 or ProS loss groups. LATE‐NC stage 3 cases were not divided into two groups because all cases had HS. As a result, p‐tau and Aβ density increased with the progression of ADNC levels. The p‐tau density in CA1 was significantly lower in the High+HS group compared to the High ADNC level group (B, *P* < 0.01), but this finding was not observed for Aβ (C). Similarly, we observed a trend toward lower pTDP‐43 density in stage 3 than in stage 2 of LATE‐NC (E, L), and lower α‐synuclein density in the Loss group than in the limbic‐neocortical type Lewy pathology group (G, N, U). However, this disparity was not statistically significant. Statistical significance was determined using the Steel–Dwass test (*P < 0.05, **P < 0.01, ***P < 0.001, ****P < 0.0001). Aβ, amyloid beta; AD, Alzheimer's disease; ADNC, Alzheimer's disease neuropathologic change; LATE‐NC, limbic‐predominant age‐related TDP‐43 encephalopathy neuropathologic change; ProS, prosubiculum; p‐tau, phosphorylated tau; pTDP‐43, phosphorylated TAR DNA‐binding protein 43.

Next, we evaluated the relationship between pyramidal cell density and accumulated protein density in disease progression in different subregions (Figure 4). As the ADNC level increased, pyramidal cell density decreased, and the area density of p‐tau and Aβ‐positive area increased significantly. The area density of the p‐tau–positive area in cases with high ADNC levels and HS was significantly lower than in cases without HS. A similar trend was observed in the pTDP‐43 area density in relation to the LATE‐NC stage and CA1 and in the area density of the α‐synuclein–positive area in cases with Lewy body pathology and CA2, although significant differences were not observed. On the other hand, the area density of the Aβ‐positive area in CA1 did not decrease when high ADNC level cases were accompanied by HS.

### 3.5 Pathological findings in each case

**FIGURE 5:**
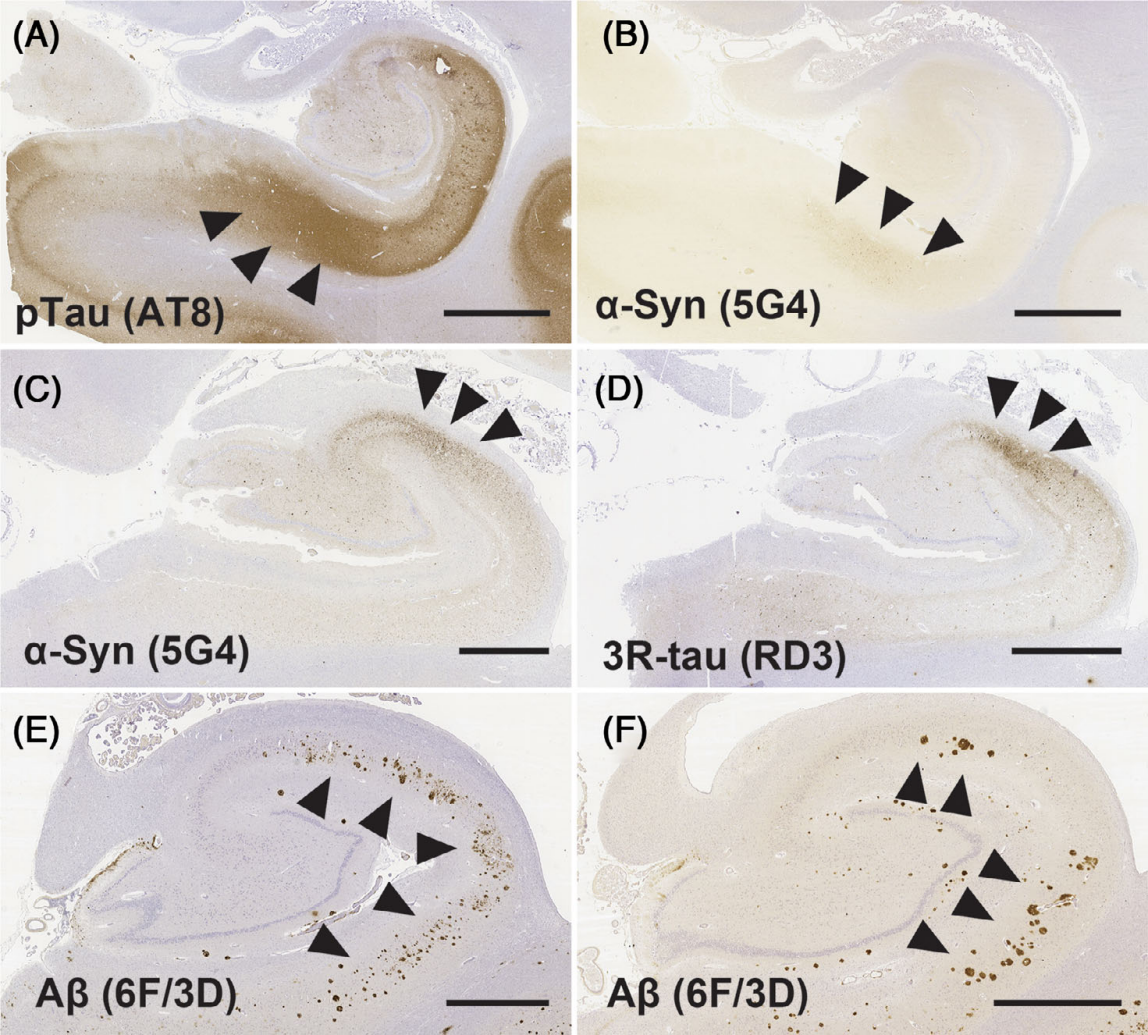
Pathological images of representative cases showing atypical distribution patterns. A and B, Sections stained for phosphorylated tau (p‐tau, AT8, A) and α‐synuclein (α‐Syn, 5G4, B) from the same case of AD+LBD. Like p‐tau, α‐synuclein also accumulates predominantly in the ProS region (arrowheads). C and D, Sections stained for α‐synuclein (C) and 3‐repeat tau (3R‐tau, RD3, D) from the same case of LBD. Both α‐synuclein and 3R‐tau predominantly accumulate in CA2 (arrowheads). Cases E and F depict separate cases of AD. In case E, Aβ (6F/3D) is distributed throughout CA1 (arrowheads), whereas in case F, there is more accumulation of Aβ in areas near CA2 and ProS (arrowheads). Scale bar: 2 mm. Aβ, amyloid beta; ProS, prosubiculum.

Observing individual cases with multiple pathologies combined, the distribution pattern of the pathology that was interpreted as less prominent appeared to be influenced by the distribution pattern of the protein pathology interpreted as predominant. For instance, α‐synuclein accumulated in ProS in AD + LBD cases with significant AD pathology (Figure 5A, B). Additionally, CA2‐predominant accumulation of p‐tau was noted in LBD cases with low levels of AD‐related pathology (Figure 5C, D). Furthermore, disseminated Aβ deposition was observed in CA1 in typical AD cases (Figure 5E), while in another case, Aβ deposition shifted to areas closer to CA2 and ProS (Figure 5F).

## 4 DISCUSSION

Evaluating 166 cases of major neurodegenerative diseases, we have delineated patterns of key protein accumulation within subregions of the hippocampus and provided a comprehensive description of how background disease and neuronal loss influence these accumulation patterns.

The pattern of hippocampal p‐tau accumulation in AD has been studied relatively extensively. Braak et al. initially observed p‐tau accumulation in the CA1 pyramidal cell layer, stratum oriens, and stratum radiatum during early AD stages.^27^ However, ProS was not identified as a distinct subregion and was not assessed separately. Subsequent studies have consistently reported CA1 predominance,^10^, ^14^, ^43^ although differences between these reports and our findings may stem mainly from annotation methods. We rigorously distinguished between the pyramidal cell layers of CA1 and ProS, potentially resulting in overestimated p‐tau accumulation in CA1 when these layers overlapped or when regions beyond the pyramidal cell layer were included in the annotation. In the CA1, p‐tau accumulates early in stratum oriens and stratum radiatum.^17^, ^27^ Moreover, as we show, p‐tau accumulates earlier in the pyramidal cell layer of ProS than in CA1, suggesting that including these sites may inflate the evaluation of p‐tau accumulation in CA1. Lace et al. identified a specific region within the ProS pyramidal cell layer as the earliest and strongest site of p‐tau accumulation in the hippocampus of AD, consistent with our findings.^17^ Remarkably, our study revealed a higher mean p‐tau accumulation in CA2 compared to CA1 in AD. While this predominance of CA2 has been sporadically reported in individual cases,^44^, ^45^ our study provides the first confirmation of this trend. The stratum oriens, in which substantial p‐tau accumulation occurs early in AD, is known to be traversed by fibers from CA2 pyramidal cells,^46^ suggesting that this pathway may be affected by p‐tau earlier than in CA1 pyramidal cells. In summary, when CA1 and ProS were evaluated separately, AD‐associated tau was as or more abundant in CA2 than in CA1, with ProS being the most abundant.

Studies on the pattern of hippocampal p‐tau accumulation in 4R tauopathies such as PSP and CBD are limited; some reports have suggested a possible predominance in CA2.^47^ However, our results from the present study did not confirm such a predominance in CA2. Our analysis evaluated the total area of p‐tau accumulation and thus could not assess each cell type separately. 4R tauopathy is characterized by tau accumulation in glial cells, such as tuft‐shaped astrocytes, and a separate evaluation of these cells may yield different results.

Few reports have compared Aβ deposition in hippocampal subregions, and none have evaluated ProS separately. However, Thal et al. reported that Aβ accumulation occurred earliest in CA1 among hippocampal subregions,^48^, ^49^ a finding that aligns with our observations. An intriguing aspect of our data is that, unlike AD‐associated tau, Aβ deposition predominantly occurred in CA1 even when evaluated separately from ProS.

The predominance of Lewy pathology–associated α‐synuclein in CA2 has been reported extensively in previous studies,^11^, ^43^, ^50^, ^51^, ^52^, ^53^ and our findings are consistent with these reports. While detailed investigations on the distribution of α‐synuclein in the hippocampus of MSA are lacking, our results suggest, for the first time, that CA2 predominance may also be present in MSA. Although α‐synuclein pathology in the hippocampus of MSA cases has garnered attention,^54^, ^55^, ^56^, ^57^ our study was limited in comprehensively analyzing overall α‐synuclein deposition in the hippocampus of MSA cases. Further investigations on the CA2 region in MSA are warranted, particularly given that previous magnetic resonance imaging studies have demonstrated an association between CA2/3 atrophy and cognitive and language functions.^58^, ^59^

The pattern of TDP‐43 accumulation in the hippocampus in LATE‐NC varied from case to case, but each group tended to have a predominance of CA1 or ProS. Previous reports have documented a predominance of TDP‐43 accumulation in CA1,^28^, ^60^, ^61^ and hippocampal sclerosis, often seen as a common complication of LATE‐NC, is defined as neuronal loss in CA1.^31^ However, these reports did not separately evaluate ProS along with other subregions. Our results, which excluded the effect of pyramidal cell loss as much as possible, indicated a possible predominance of CA1 and ProS in LATE‐NC.

**FIGURE 6:**
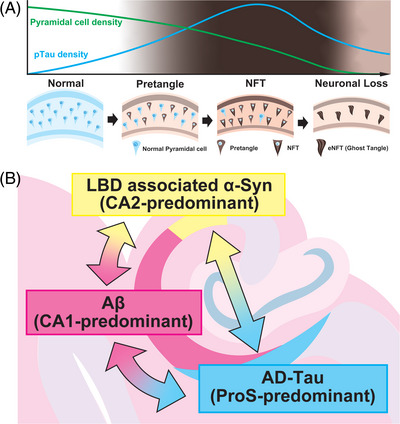
Illustrations to explain each phenomenon; (A) illustrates that the density of p‐tau in the CA1 region associated with AD increases with disease progression, whereas the density of pyramidal cells gradually decreases. This rise in p‐tau density reaches a peak level and subsequently declines, demonstrating a “peak‐out” phenomenon; (B) shows how p‐tau associated with AD, Aβ, and α‐synuclein associated with Lewy body disease exhibit predominance in ProS, CA1, and CA2, respectively. Furthermore, the distribution of each protein is influenced by the presence of other proteins. Aβ, amyloid beta; AD, Alzheimer's disease; LBD, Lewy body disease; NFT, neurofibrillary tangle; ProS, prosubiculum; p‐tau, phosphorylated tau.

We were the first to demonstrate decreased accumulated protein with neuronal loss in the final stages of disease progression (Figure 6A). Many neuropathologists would have observed this phenomenon but did not attempt to prove it. Our results confirmed it for p‐tau, pTDP‐43, and α‐synuclein. This phenomenon highlights the risk of assessing disease progression solely based on the amount of accumulated protein. This is particularly relevant for pathologies such as LATE‐NC, in which neurons are prone to loss, and we evaluated pTDP‐43 distribution in LATE‐NC cases, excluding cases with severe pyramidal cell loss such as in HS.

Very interestingly, for the first time, we have comprehensively shown that additional protein depositions influence the distribution pattern of the leading protein accumulation in each proteinopathy. Specifically, AD‐associated p‐tau predominantly accumulates in ProS, Aβ in CA1, and α‐synuclein in LBD accumulates in CA2, each of which may impact the distribution of another accumulated protein (Figure 6B). Some aspects of this phenomenon have been reported previously: the observed pattern of AD‐associated tau being stronger in CA2/3 than in CA1/Sub in LBD^43^, ^53^ was not statistically significant in our results, but a similar trend was observed, and cases in which this pattern was typical were also identified. It has been reported that p‐tau in PART is mainly deposited in CA2 and shifts to CA1 predominance in AD,^14^, ^62^ which may be a change due to the influence from Aβ we observed. Additionally, a study on protein distribution throughout the brain indicates that LATE‐NC distribution in AD differs from that in LBD.^28^ It is conceivable that the interaction in the distribution of these proteinopathies extends beyond the hippocampus, and further investigations involving a broader range of proteinopathies are warranted.

## 5 LIMITATIONS

The first limitation of this study is the bias inherent in our cohort. Because this is a pathology‐based cohort rather than a community cohort, it does not include an equal distribution of mild to severe cases, and there is a bias toward severe cases of each disease. The cohort is also skewed toward elderly individuals and the clinical information from the archival cases does not allow us to perform systematic clinicopathological correlations. Furthermore, the number of cases was insufficient for a comprehensive evaluation of hippocampal subregions across some proteinopathies. This, coupled with the challenge of using neuropathological assessment methods that rely on pathological stages that are statistically difficult to manage, made the evaluation challenging. Nevertheless, we attempted to mitigate these limitations by including as many cases as possible and conducting regression analyses along with sensitivity analyses.

The second limitation is related to the evaluation of pyramidal cell loss, which was assessed by manual counting within limited regions. However, manual counting across the entire region would require counting thousands of cells in the CA1 region alone for each case, making it impractical. Automatic counting by image analysis software, even with sufficient adjustment, does not provide the required accuracy. To address this issue, we evaluated a new method and validated its consistency with automatic counting in some cases (Figure S2).

To overcome these limitations in future studies, evaluations in larger or community‐based cohorts that include younger cases and the use of new methods, such as artificial intelligence, that address both accuracy and manpower constraints may prove effective.

## 6 CONCLUSIONS

We comprehensively evaluated protein accumulation across various subregions of the hippocampus, including ProS, in a cohort of 166 cases representing diverse neurodegenerative diseases. Our analysis revealed distinctive patterns in each major degenerative disease. Specifically, we confirmed that AD‐related tau predominantly accumulated in ProS, Aβ in CA1, and LBD‐related α‐synuclein in CA2. Furthermore, we observed, for the first time, an increase in the accumulation of each protein with disease progression, which subsequently decreased during late‐stage disease progression characterized by pyramidal cell loss. Additionally, we provided evidence suggesting that the accumulation patterns of p‐tau, Aβ, and α‐synuclein in the hippocampus may be mutually influenced by comorbid pathologies.

## CONFLICT OF INTEREST STATEMENT

GGK has served as an advisor for Biogen in 2019 and Parexel in 2023; received a royalty for 5G4 synuclein antibody and publishing royalties from Wiley, Cambridge University Press, and Elsevier; received grants from Edmond J. Safra Philanthropic Foundation, Rossy Family Foundation, Michael J. Fox Foundation, Krembil Foundation, MSA Coalition, NIH, Parkinson Canada, Canada, and Canada Foundation for Innovation. SLF is supported by the National Health and Medical Research Council of Australia Ideas grant (#214090508). These have no relevance for this study. KY, SI, HT, and TK declare no competing interests. Author disclosures are available in the supporting information.

## CONSENT STATEMENT

All brain specimens were approved by the UHN Research Ethics Committee (No. 20‐5258) and the University of Toronto (No. 39459) and performed according to the ethical standards outlined in the 1964 Declaration of Helsinki (updated 2008).

## Supporting information



Supporting Information

Supporting Information
