# Supplementary material for: Co‐pathologies modify hippocampal protein accumulation patterns in neurodegenerative diseases
Source: Alzheimers Dement. 2024 Dec 23;21(1):e14355. doi: 10.1002/alz.14355 (PMC11782183; doi:10.1002/alz.14355)
Supplement: Supplementary file 1 — Supporting Information [file ALZ-21-e14355-s002.pdf]

Co-pathologies modify hippocampal protein accumulation patterns in neurodegenerative diseases

Koji Yoshida[1,2,3], Shelley L. Forrest[1,2,4,5], Shojiro Ichimata[1,2,3], Hidetomo Tanaka[1,2], Tomoya Kon[1,2,6], Gabor G. Kovacs[1,2,4,5]

1. Department of Laboratory Medicine and Pathobiology and Department of Medicine, University of Toronto, Toronto, ON, Canada
2. Tanz Centre for Research in Neurodegenerative Disease, Krembil Discovery Tower, University of Toronto, 60 Leonard Ave Toronto On, Toronto, ON M5T 0S8, Canada
3. Department of Legal Medicine, Graduate School of Medicine and Pharmaceutical Sciences, University of Toyama, Toyama, Japan.
4. Laboratory Medicine Program & Krembil Brain Institute, University Health Network, Toronto, ON, Canada
5. Department of Neurology, Dementia Research Centre, Macquarie Medical School, Faculty of Medicine, Health and Human Sciences, Macquarie University, Sydney, Australia
6. Graduate School of Medicine, Hirosaki, University, Aomori, Japan

Supplemental Table 1. Regression analysis (ANCOVA) for protein comparison scores using the pyramidal cell density

Explanatory variable (target side)	CA1-CA2 Score															
	pTau (AT8) CA1-2 Score				Aβ CA1-2 Score				α-Synuclein (5G4) CA1-2 Score				pTDP-43 CA1-2 Score			
	Estimated value	95%CI		P value	Estimated value	95%CI		P value	Estimated value	95%CI		P value	Estimated value	95%CI		P value
**Braak NFT Stage** (high)	**-0.128**	**-0.239**	**-0.017**	**0.0242**	-0.002	-0.142	0.139	0.9794	0.010	-0.065	0.085	0.7997	0.019	-0.062	0.100	0.6450
**Thal Phase** (high)	**0.123**	**0.014**	**0.233**	**0.0278**	**0.142**	**0.003**	**0.281**	**0.0447**	0.036	-0.039	0.110	0.3442	-0.043	-0.123	0.038	0.2931
**Lewy Pathology Type** (severe)	-0.018	-0.089	0.053	0.6160	-0.086	-0.175	0.003	0.0568	**-0.270**	**-0.317**	**-0.223**	**<0.0001**	-0.027	-0.078	0.024	0.2960
**AGD Saito Stage** (high)	-0.003	-0.098	0.093	0.9556	0.036	-0.082	0.155	0.5462	-0.010	-0.074	0.053	0.7429	0.001	-0.067	0.070	0.9739
**LATE-NC Stage** (high)	-0.037	-0.135	0.060	0.4465	-0.026	-0.150	0.097	0.6709	-0.027	-0.093	0.039	0.4151	**0.110**	**0.038**	**0.181**	**0.0029**
**CA1 Pyramidal Cell Density** (/mm2)	0.001	-0.001	0.003	0.3875	**-0.003**	**-0.006**	**0.000**	**0.0400**	0.000	-0.001	0.002	0.5873	0.000	-0.002	0.002	0.9196
**CA2 Pyramidal Cell Density** (/mm2)	0.000	-0.002	0.001	0.5216	0.000	-0.002	0.002	0.8753	0.001	0.000	0.002	0.2110	-0.001	-0.002	0.001	0.3043

Explanatory variable (target side)	CA1-ProS Score															
	pTau (AT8) CA1-ProS Score				Aβ CA1-ProS Score				α-Synuclein (5G4) CA1-ProS Score				pTDP-43 CA1-ProS Score			
	Estimated value	95%CI		P value	Estimated value	95%CI		P value	Estimated value	95%CI		P value	Estimated value	95%CI		P value
**Braak NFT Stage** (high)	-0.024	-0.109	0.061	0.5747	0.006	-0.134	0.146	0.9325	-0.033	-0.105	0.039	0.3693	-0.032	-0.095	0.031	0.3161
**Thal Phase** (high)	**-0.098**	**-0.182**	**-0.013**	**0.0240**	**0.152**	**0.012**	**0.291**	**0.0333**	-0.006	-0.078	0.066	0.8685	0.025	-0.038	0.088	0.4289
**Lewy Pathology Type** (severe)	-0.047	-0.099	0.006	0.0795	-0.081	-0.167	0.006	0.0663	**-0.094**	**-0.138**	**-0.050**	**<0.0001**	-0.012	-0.051	0.027	0.5450
**AGD Saito Stage** (high)	**-0.073**	**-0.145**	**-0.002**	**0.0444**	0.029	-0.086	0.145	0.6135	0.042	-0.018	0.101	0.1671	0.004	-0.049	0.056	0.8901
**LATE-NC Stage** (high)	0.064	-0.013	0.140	0.1018	-0.005	-0.131	0.122	0.9394	**0.070**	**0.004**	**0.135**	**0.0369**	**-0.089**	**-0.148**	**-0.030**	**0.0033**
**CA1 Pyramidal Cell Density** (/mm2)	0.000	-0.001	0.002	0.6882	**-0.004**	**-0.007**	**-0.001**	**0.0093**	-0.001	-0.002	0.001	0.3989	-0.001	-0.002	0.001	0.4460
**ProS Pyramidal Cell Density** (/mm2)	-0.001	-0.003	0.000	0.1348	0.001	-0.001	0.004	0.2752	**0.001**	**0.000**	**0.003**	**0.0411**	-0.001	-0.002	0.001	0.2301

Explanatory variable (target side)	CA2-ProS Score															
	pTau (AT8) CA2-ProS Score				Aβ CA2-ProS Score				α-Synuclein (5G4) CA2-ProS Score				pTDP-43 CA2-ProS Score			
	Estimated value	95%CI		P value	Estimated value	95%CI		P value	Estimated value	95%CI		P value	Estimated value	95%CI		P value
**Braak NFT Stage** (high)	0.078	-0.049	0.205	0.2282	-0.033	-0.199	0.133	0.6925	-0.054	-0.147	0.039	0.2551	-0.019	-0.102	0.064	0.6557
**Thal Phase** (high)	**-0.192**	**-0.318**	**-0.065**	**0.0034**	-0.060	-0.225	0.106	0.4746	-0.031	-0.124	0.062	0.5115	0.044	-0.038	0.127	0.2915
**Lewy Pathology Type** (severe)	-0.024	-0.103	0.055	0.5472	**0.105**	**0.003**	**0.208**	**0.0444**	**0.176**	**0.119**	**0.233**	**<0.0001**	0.016	-0.034	0.067	0.5243
**AGD Saito Stage** (high)	-0.064	-0.171	0.043	0.2414	-0.055	-0.192	0.082	0.4266	0.032	-0.045	0.109	0.4159	0.015	-0.053	0.084	0.6540
**LATE-NC Stage** (high)	0.096	-0.020	0.212	0.1036	-0.046	-0.198	0.106	0.5482	**0.110**	**0.025**	**0.195**	**0.0118**	**-0.147**	**-0.223**	**-0.071**	**0.0002**
**CA2 Pyramidal Cell Density** (/mm2)	0.000	-0.001	0.002	0.7712	0.001	-0.001	0.004	0.1853	-0.001	-0.002	0.000	0.0776	0.001	0.000	0.002	0.0702
**ProS Pyramidal Cell Density** (/mm2)	-0.001	-0.004	0.001	0.3517	0.001	-0.003	0.004	0.6792	0.001	-0.001	0.003	0.2771	-0.001	-0.002	0.001	0.4904

Abbreviation: *ANCOVA* Analysis of covariance; *Aβ* Amyloid β; *pTDP-43* phosphorylated Transactive response DNA binding protein of 43kDa; *NFT* Neurofibrillary tangle; *LBD* Lewy body pathology; *AGD* Argyrophilic Grain Disease; *LATE* Limbic-predominant age-related TDP-43 encephalopathy.

Supplemental Table 2. Regression analysis (ANCOVA) for protein comparison scores using age, sex, and vessel score.

Explanatory variable (target side)	CA1-CA2 Score															
	**pTau (AT8) CA1-2 Score**				**Aβ CA1-2 Score**				**α-Synuclein (5G4) CA1-2 Score**				**pTDP-43 CA1-2 Score**			
	Estimated value	95%CI		P value	Estimated value	95%CI		P value	Estimated value	95%CI		P value	Estimated value	95%CI		P value
**Age**	-0.002	-0.009	0.004	0.4364	-0.001	-0.009	0.007	0.7842	**-0.005**	**-0.008**	**-0.001**	**0.0258**	0.005	0.001	0.010	0.0138
**Sex** (female)	-0.011	-0.081	0.059	0.7583	-0.001	-0.091	0.089	0.9816	**0.052**	**0.007**	**0.097**	**0.0246**	-0.010	-0.059	0.040	0.6948
**Vessel Score** (high)	-0.002	-0.068	0.063	0.9452	0.038	-0.047	0.123	0.3754	0.021	-0.022	0.064	0.3364	-0.007	-0.054	0.039	0.7505
**Braak NFT Stage** (high)	**-0.127**	**-0.238**	**-0.016**	**0.0254**	0.026	-0.118	0.169	0.7246	-0.013	-0.085	0.060	0.7329	0.026	-0.053	0.105	0.5152
**Thal Phase** (high)	**0.133**	**0.021**	**0.245**	**0.0203**	**0.149**	**0.003**	**0.294**	**0.0449**	0.049	-0.025	0.122	0.1900	-0.060	-0.140	0.021	0.1436
**Lewy Pathology Type** (severe)	-0.008	-0.078	0.062	0.8203	**-0.104**	**-0.194**	**-0.014**	**0.0237**	**-0.266**	**-0.311**	**-0.220**	**<0.0001**	-0.028	-0.078	0.021	0.2564
**AGD Saito Stage** (high)	0.026	-0.067	0.118	0.5851	0.000	-0.116	0.117	0.9936	0.012	-0.047	0.071	0.6879	-0.020	-0.085	0.044	0.5347
**LATE-NC Stage** (high)	-0.046	-0.135	0.043	0.3042	0.038	-0.076	0.153	0.5084	-0.034	-0.092	0.024	0.2436	**0.104**	**0.040**	**0.167**	**0.0016**

Explanatory variable (target side)	CA1-ProS Score															
	**pTau (AT8) CA1-ProS Score**				**Aβ CA1-ProS Score**				**α-Synuclein (5G4) CA1-ProS Score**				**pTDP-43 CA1-ProS Score**			
	Estimated value	95%CI		P value	Estimated value	95%CI		P value	Estimated value	95%CI		P value	Estimated value	95%CI		P value
**Age**	0.000	-0.005	0.005	0.9540	0.002	-0.005	0.009	0.5854	-0.001	-0.005	0.003	0.4847	0.000	-0.004	0.003	0.9838
**Sex** (female)	-0.021	-0.077	0.035	0.4549	0.017	-0.064	0.098	0.6856	0.020	-0.025	0.065	0.3845	0.015	-0.025	0.055	0.4549
**Vessel Score** (high)	-0.023	-0.076	0.029	0.3761	0.000	-0.076	0.077	0.9957	0.019	-0.024	0.062	0.3725	-0.008	-0.045	0.030	0.6895
**Braak NFT Stage** (high)	-0.012	-0.101	0.077	0.7953	-0.065	-0.195	0.064	0.3194	-0.045	-0.118	0.028	0.2247	-0.024	-0.087	0.040	0.4612
**Thal Phase** (high)	**-0.098**	**-0.188**	**-0.008**	**0.0324**	0.071	-0.060	0.202	0.2875	-0.010	-0.083	0.064	0.7935	0.031	-0.033	0.096	0.3357
**Lewy Pathology Type** (severe)	-0.050	-0.105	0.006	0.0813	-0.005	-0.086	0.076	0.8949	**-0.099**	**-0.145**	**-0.054**	**<0.0001**	-0.011	-0.051	0.029	0.5866
**AGD Saito Stage** (high)	**-0.086**	**-0.161**	**-0.012**	**0.0228**	-0.101	-0.206	0.005	0.0609	0.057	-0.002	0.117	0.0578	-0.014	-0.066	0.038	0.5860
**LATE-NC Stage** (high)	**0.092**	**0.021**	**0.163**	**0.0118**	0.030	-0.073	0.134	0.5656	0.052	-0.006	0.111	0.0769	**-0.057**	**-0.108**	**-0.006**	**0.0289**

Explanatory variable (target side)	CA2-ProS Score															
	**pTau (AT8) CA2-ProS Score**				**Aβ CA2-ProS Score**				**α-Synuclein (5G4) CA2-ProS Score**				**pTDP-43 CA2-ProS Score**			
	Estimated value	95%CI		P value	Estimated value	95%CI		P value	Estimated value	95%CI		P value	Estimated value	95%CI		P value
**Age**	0.001	-0.006	0.008	0.7945	0.001	-0.008	0.011	0.7458	0.004	-0.002	0.009	0.1740	**-0.005**	**-0.010**	**-0.001**	**0.0198**
**Sex** (female)	0.002	-0.080	0.085	0.9593	0.024	-0.079	0.127	0.6458	-0.027	-0.085	0.031	0.3519	0.017	-0.034	0.068	0.5039
**Vessel Score** (high)	-0.015	-0.093	0.062	0.6935	-0.060	-0.158	0.038	0.2244	-0.014	-0.069	0.041	0.6074	0.006	-0.042	0.054	0.8071
**Braak NFT Stage** (high)	0.088	-0.043	0.219	0.1841	-0.061	-0.227	0.104	0.4642	-0.045	-0.138	0.048	0.3423	-0.027	-0.108	0.054	0.5140
**Thal Phase** (high)	**-0.176**	**-0.308**	**-0.044**	**0.0094**	-0.077	-0.244	0.090	0.3643	-0.045	-0.140	0.049	0.3403	0.062	-0.020	0.145	0.1359
**Lewy Pathology Type** (severe)	-0.018	-0.101	0.064	0.6620	0.102	-0.001	0.206	0.0526	**0.180**	**0.122**	**0.238**	**<0.0001**	0.015	-0.036	0.066	0.5595
**AGD Saito Stage** (high)	-0.080	-0.189	0.029	0.1500	-0.065	-0.199	0.070	0.3404	0.036	-0.039	0.112	0.3435	0.025	-0.042	0.091	0.4615
**LATE-NC Stage** (high)	**0.121**	**0.017**	**0.226**	**0.0234**	-0.078	-0.211	0.054	0.2414	**0.089**	**0.014**	**0.163**	**0.0199**	**-0.130**	**-0.195**	**-0.065**	**0.0001**

Abbreviation: *ANCOVA* Analysis of covariance; *Aβ* Amyloid β; *pTDP-43* phosphorylated Transactive response DNA binding protein of 43kDa; *NFT* Neurofibrillary tangle; *LBD* Lewy body pathology; *AGD* Argyrophilic Grain Disease; *LATE* Limbic-predominant age-related TDP-43 encephalopathy.

Supplemental Table 3. Regression analysis (ANCOVA) for protein comparison scores using the genetic factors

Explanatory variable (target side)	CA1-CA2 Score															
	pTau (AT8) CA1-2 Score				Aβ CA1-2 Score				α-Synuclein (5G4) CA1-2 Score				pTDP-43 CA1-2 Score			
	Estimated value	95%CI		P value	Estimated value	95%CI		P value	Estimated value	95%CI		P value	Estimated value	95%CI		P value
**Braak NFT Stage** (high)	-0.092	-0.237	0.053	0.2069	-0.060	-0.260	0.141	0.5498	0.024	-0.117	0.164	0.7349	0.019	-0.132	0.170	0.8032
**Thal Phase** (high)	0.033	-0.154	0.221	0.7199	0.068	-0.193	0.329	0.6025	0.055	-0.128	0.237	0.5496	-0.129	-0.326	0.068	0.1925
**Lewy Pathology Type** (severe)	-0.024	-0.183	0.134	0.7599	**-0.209**	**-0.416**	**-0.003**	**0.0474**	**-0.246**	**-0.391**	**-0.102**	**0.0013**	-0.041	-0.197	0.115	0.5970
**AGD Saito Stage** (high)	-0.008	-0.122	0.107	0.8914	-0.116	-0.273	0.041	0.1426	0.001	-0.109	0.111	0.9890	-0.036	-0.154	0.083	0.5474
**LATE-NC Stage** (high)	-0.030	-0.136	0.076	0.5707	0.060	-0.086	0.205	0.4119	-0.070	-0.172	0.032	0.1725	0.076	-0.034	0.185	0.1708
***APOE* ε4 (positive)**	-0.006	-0.127	0.114	0.9142	-0.034	-0.196	0.127	0.6692	-0.040	-0.153	0.073	0.4756	-0.003	-0.124	0.119	0.9668
***MAPT* H1/H1 (positive)**	0.033	-0.069	0.135	0.5186	-0.074	-0.215	0.067	0.2965	-0.018	-0.117	0.081	0.7128	-0.039	-0.146	0.067	0.4579

Explanatory variable (target side)	CA1-ProS Score															
	pTau (AT8) CA1-ProS Score				Aβ CA1-ProS Score				α-Synuclein (5G4) CA1-ProS Score				pTDP-43 CA1-ProS Score			
	Estimated value	95%CI		P value	Estimated value	95%CI		P value	Estimated value	95%CI		P value	Estimated value	95%CI		P value
**Braak NFT Stage** (high)	-0.003	-0.109	0.104	0.9610	**-0.194**	**-0.371**	**-0.017**	**0.0326**	**-0.123**	**-0.228**	**-0.018**	**0.0230**	-0.027	-0.113	0.058	0.5248
**Thal Phase** (high)	-0.075	-0.214	0.063	0.2778	0.125	-0.106	0.355	0.2823	-0.069	-0.207	0.068	0.3135	0.008	-0.103	0.119	0.8849
**Lewy Pathology Type** (severe)	0.004	-0.113	0.121	0.9447	-0.067	-0.250	0.116	0.4623	**-0.142**	**-0.250**	**-0.033**	**0.0117**	-0.005	-0.093	0.083	0.9150
**AGD Saito Stage** (high)	-0.056	-0.140	0.029	0.1892	-0.118	-0.257	0.021	0.0933	0.010	-0.072	0.093	0.8031	-0.040	-0.106	0.027	0.2394
**LATE-NC Stage** (high)	**0.083**	**0.005**	**0.160**	**0.0381**	0.003	-0.126	0.131	0.9670	0.064	-0.012	0.141	0.0965	**-0.121**	**-0.183**	**-0.059**	**0.0003**
***APOE* ε4 (positive)**	0.067	-0.022	0.156	0.1354	-0.060	-0.203	0.082	0.3986	0.029	-0.056	0.113	0.4998	0.031	-0.038	0.100	0.3713
***MAPT* H1/H1 (positive)**	-0.018	-0.093	0.056	0.6194	-0.025	-0.150	0.099	0.6823	-0.018	-0.092	0.056	0.6322	-0.024	-0.084	0.036	0.4299

Explanatory variable (target side)	CA2-ProS Score															
	pTau (AT8) CA2-ProS Score				Aβ CA2-ProS Score				α-Synuclein (5G4) CA2-ProS Score				pTDP-43 CA2-ProS Score			
	Estimated value	95%CI		P value	Estimated value	95%CI		P value	Estimated value	95%CI		P value	Estimated value	95%CI		P value
**Braak NFT Stage** (high)	0.050	-0.123	0.224	0.5618	-0.042	-0.264	0.180	0.7052	-0.143	-0.301	0.016	0.0769	-0.020	-0.176	0.136	0.8006
**Thal Phase** (high)	-0.102	-0.326	0.123	0.3645	-0.028	-0.317	0.261	0.8439	-0.103	-0.309	0.104	0.3228	0.091	-0.112	0.294	0.3707
**Lewy Pathology Type** (severe)	0.033	-0.156	0.223	0.7243	0.117	-0.112	0.346	0.3088	0.126	-0.038	0.290	0.1277	0.021	-0.140	0.181	0.7969
**AGD Saito Stage** (high)	-0.043	-0.181	0.094	0.5260	0.006	-0.168	0.180	0.9459	0.017	-0.108	0.141	0.7871	0.023	-0.099	0.145	0.7037
**LATE-NC Stage** (high)	0.097	-0.030	0.225	0.1298	-0.120	-0.281	0.041	0.1391	**0.138**	**0.023**	**0.253**	**0.0202**	**-0.157**	**-0.270**	**-0.044**	**0.0076**
***APOE* ε4 (positive)**	0.074	-0.070	0.219	0.3043	0.034	-0.145	0.213	0.7034	0.060	-0.067	0.188	0.3462	0.021	-0.105	0.147	0.7376
***MAPT* H1/H1 (positive)**	-0.061	-0.184	0.061	0.3166	0.058	-0.098	0.214	0.4581	-0.010	-0.122	0.102	0.8565	0.000	-0.110	0.110	0.9982

Abbreviation: *ANCOVA* Analysis of covariance; *Aβ* Amyloid β; *pTDP-43* phosphorylated Transactive response DNA binding protein of 43kDa; *NFT* Neurofibrillary tangle; *LBD* Lewy body pathology; *AGD* Argyrophilic Grain Disease; *LATE* Limbic-predominant age-related TDP-43 encephalopathy.

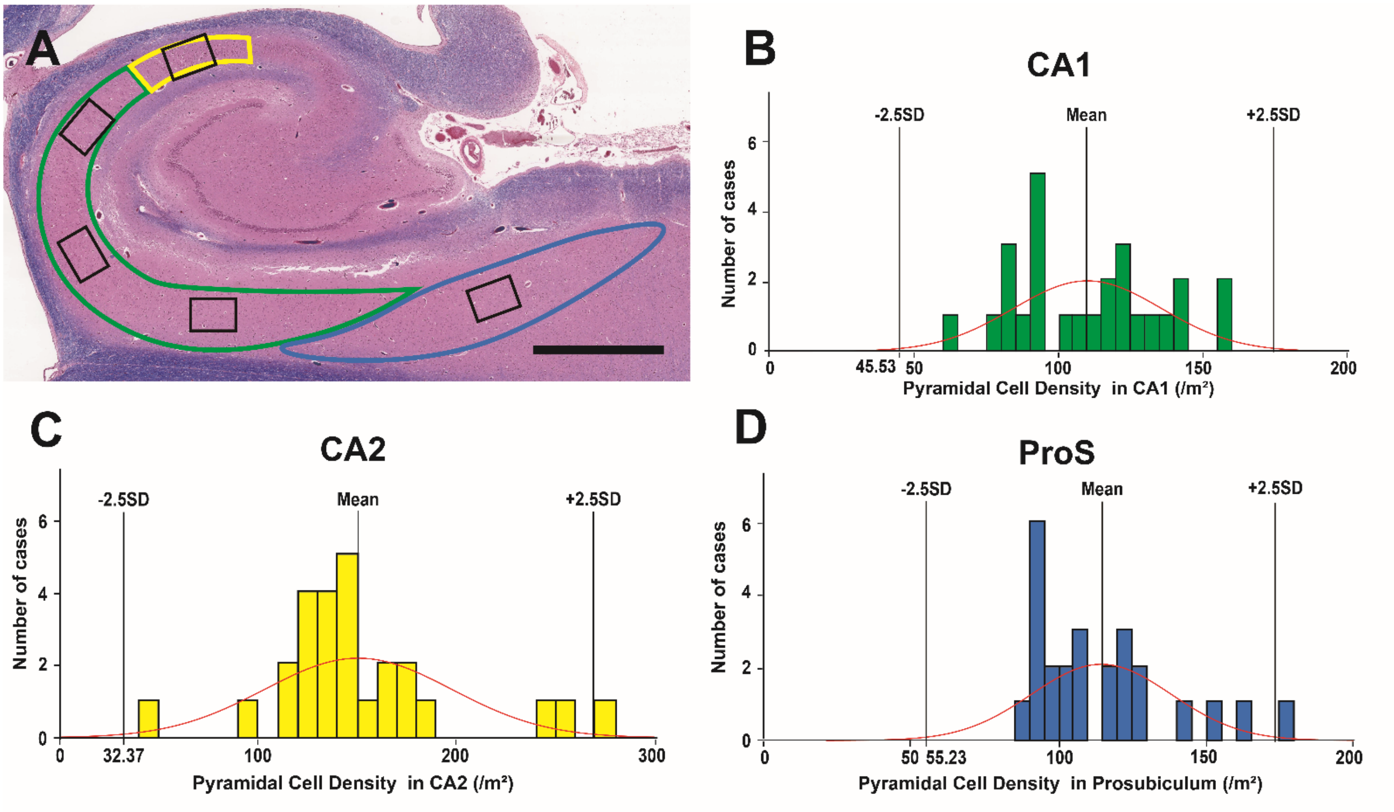

Supplemental Figure 1. Measurement of pyramidal cell density and establishment of neuronal loss threshold

A shows an example of pyramidal cell density measurement. For each subregion of CA1 (green), CA2 (yellow), and Prosubiculum (ProS, blue) in all cases, three locations for CA1 and one location each for CA2 and ProS were randomly photographed (black squares) in the 100x field of view, and the pyramidal cell density within the photographed area was measured. The pyramidal cell density of each subregion in control cases was then plotted, with a cutoff criterion of less than -2.5 SD for each subregion (B: CA1, C: CA2, D: ProS).

Scale bar: 1mm (A)

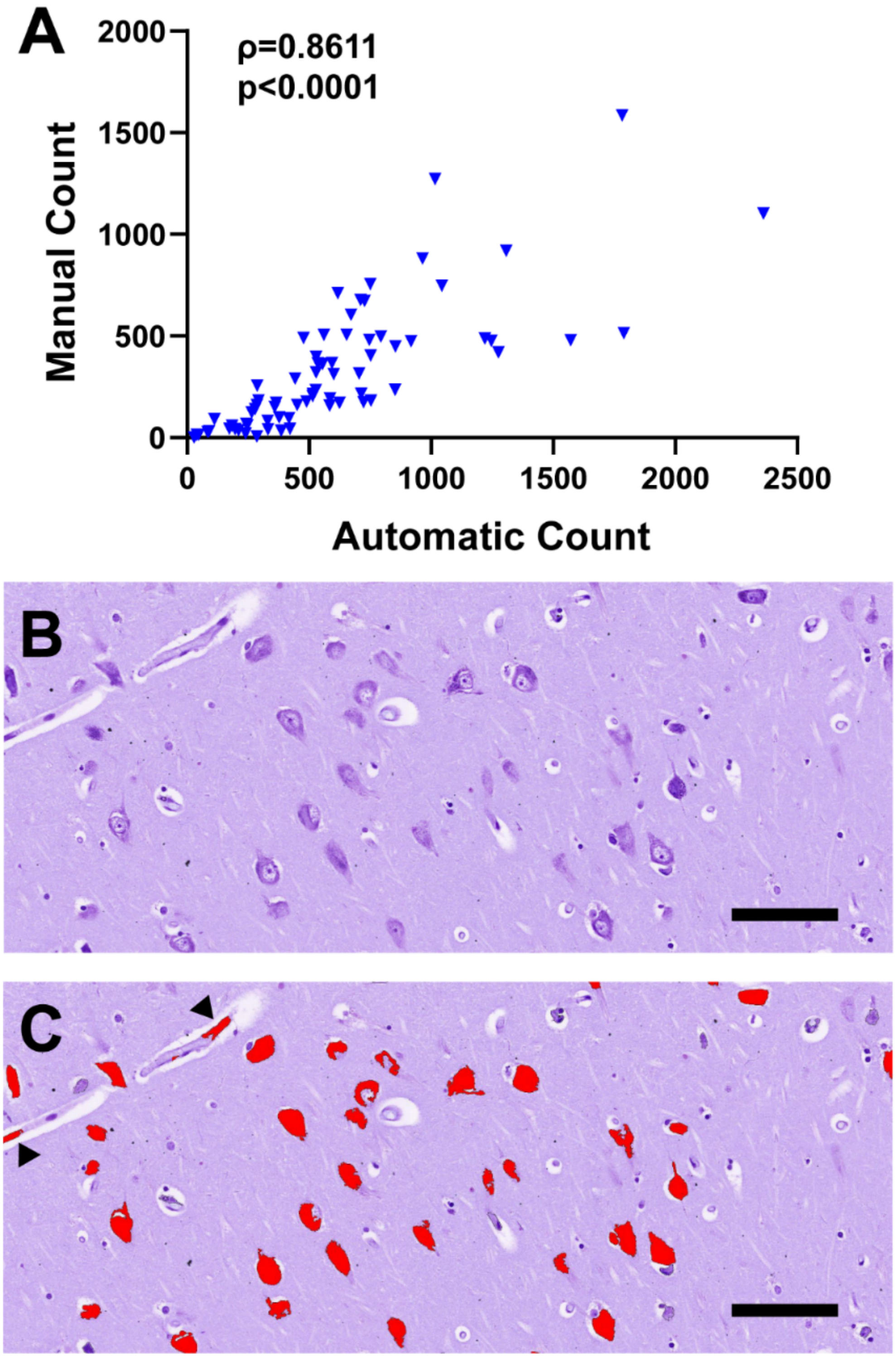

Supplemental Figure 2. Measurement of Pyramidal Cell Counts Using Image Analysis Software.

The correlation between the manual counts used in this study and the automated counts performed using image analysis software (Halo) and Nissl-stained sections showed a robust correlation (ρ=0.8611, p<0.0001, Spearman's correlation coefficient). Still, the automated counts tended to be more frequent (A). Comparing the pre-annotated image (B) and the post-annotated image (C) used in automatic counting, it can be seen that automatic counting also detected some blood vessels in the count (arrowheads).

Scale bar: 100μm (b-c)

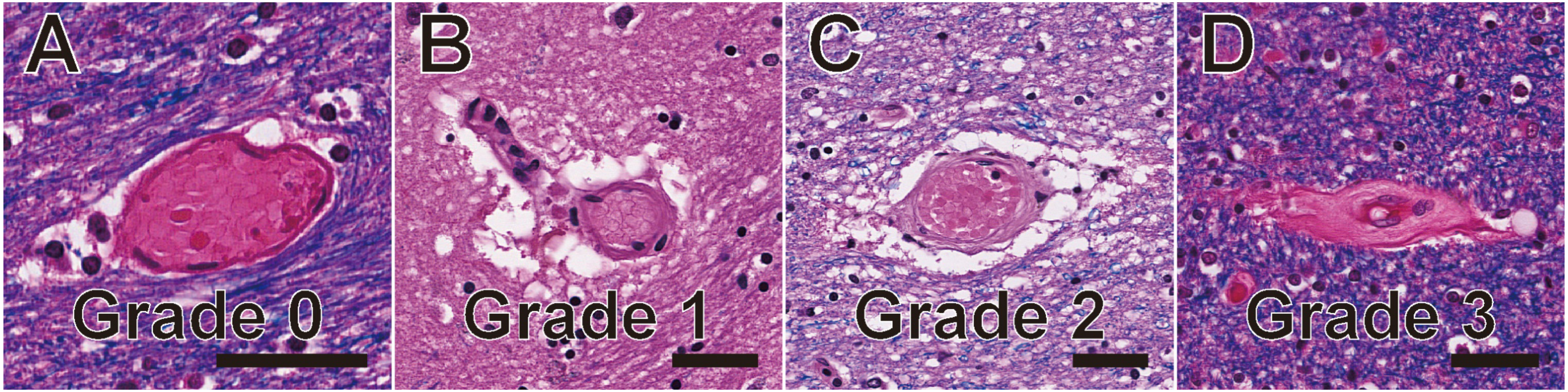

Supplemental Figure 3. Vascular wall thickening grading

Vascular wall thickening was graded according to the Vascular Cognitive Impairment Neuropathology Guidelines (VCING). The grading was as follows: Normal vessels were assigned Grade 0 (A), vessels with thickened walls but no lipohyalinosis were assigned Grade 1 (B), vessels with lipohyalinosis but no obvious lumen narrowing were assigned Grade 3 (C), and vessels with obvious lumen narrowing were assigned Grade 4 (D).

Scale bar: 40μm

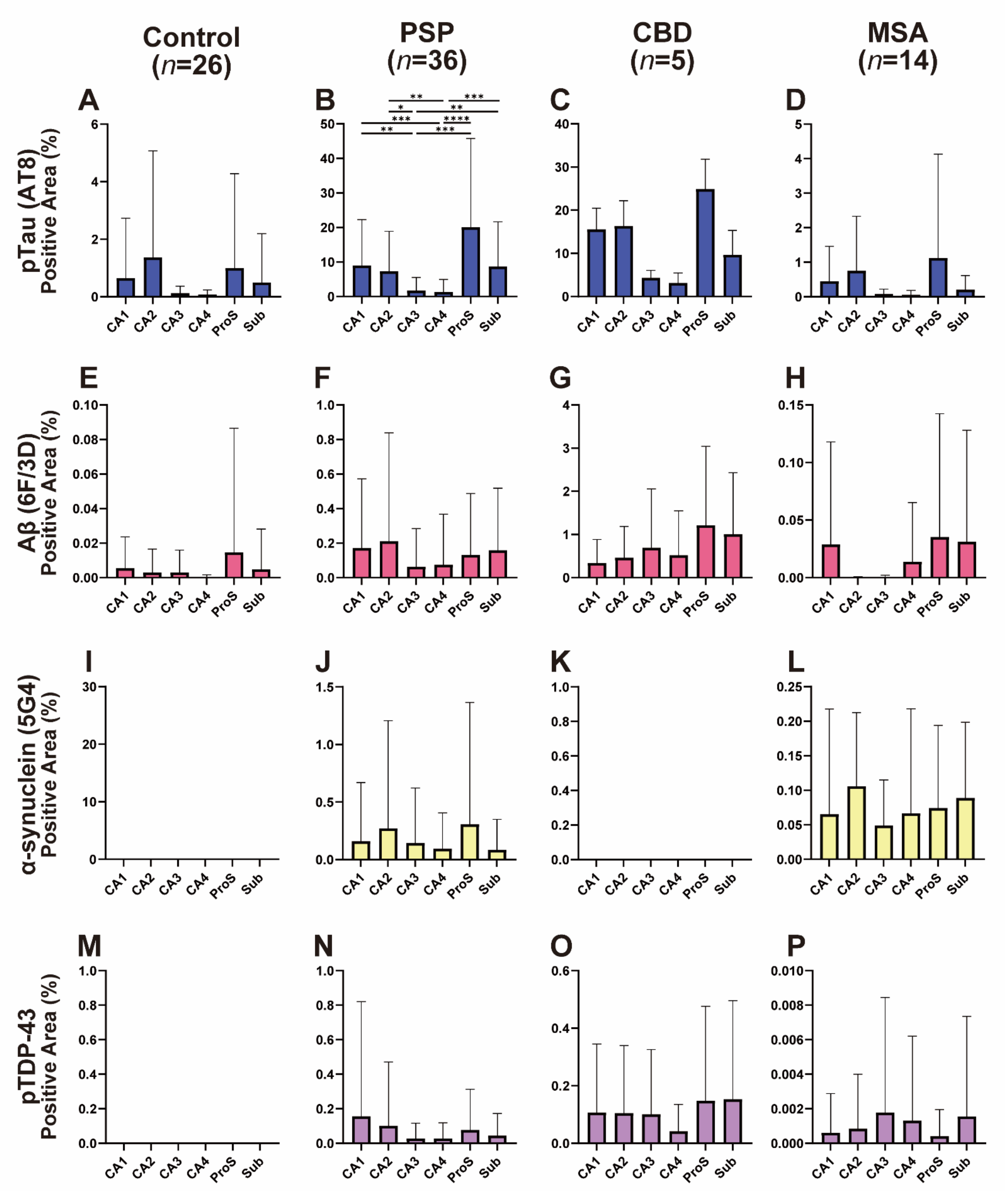

Supplemental Figure 4. Protein accumulation in each subregion of the control, PSP, CBD, and MSA groups

The control (A, E, I, M), PSP (B, F, J, N), CBD (C, G, K, O), MSA (D, H, L, P) were assessed for accumulation of pTau (AT8, A-D), Aβ (6F/3D, E-H), α-synuclein (5G4, I-L), and pTDP-43 (M-P) in each subregion of the hippocampus. Statistical significance was determined using the Steel-Dwass test (*p<0.05, **p<0.01, ***p<0.001, ****p<0.0001).

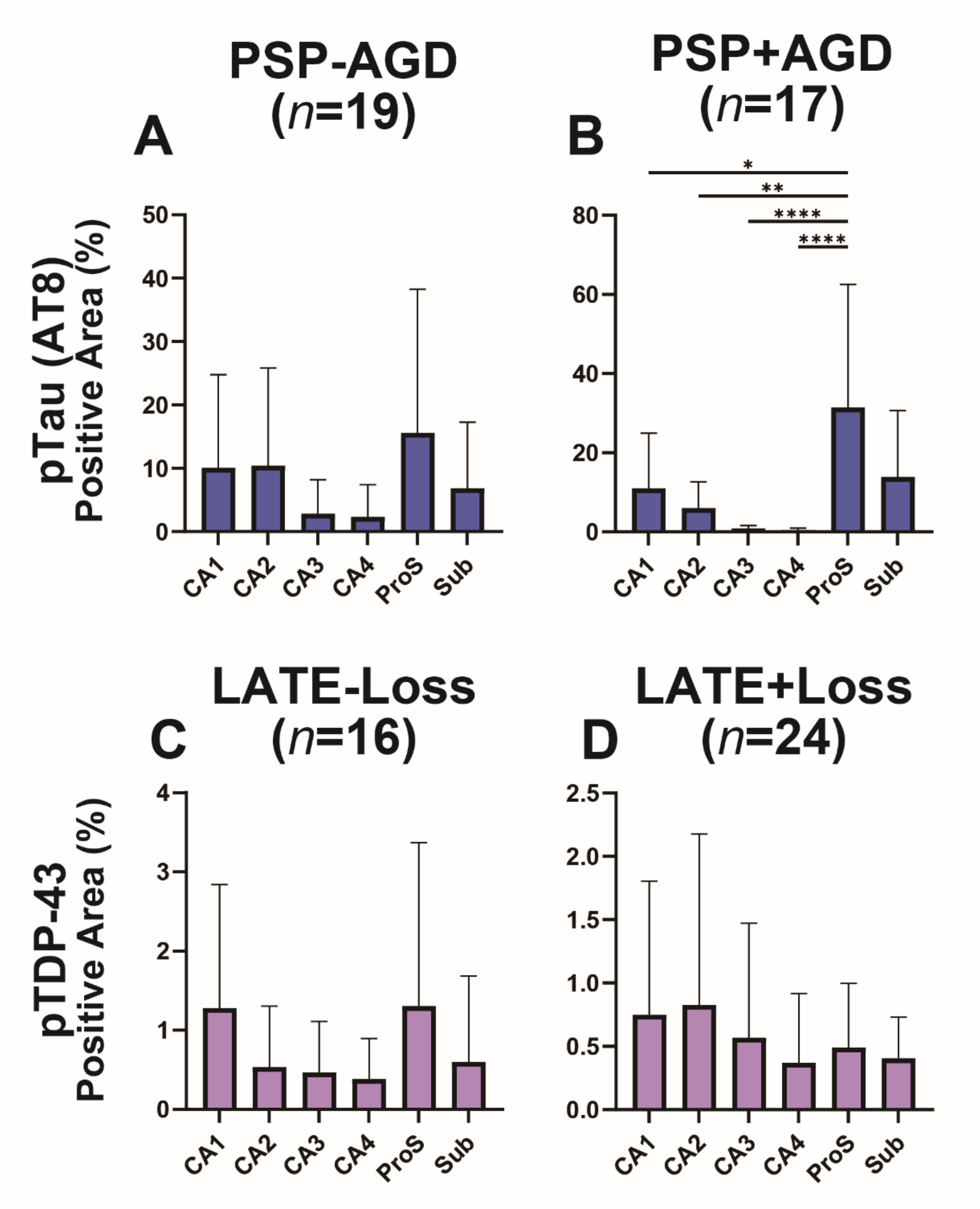

Supplemental Figure 5. Additional analysis of protein accumulation in each subregion for PSP and LATE-NC

Additional analysis of pTau and pTDP-43 subregion accumulation was performed for cases with PSP pathology (A, B) and LATE-NC (C, D), respectively. pTau accumulation was highest in ProS both in PSP cases with AGD (A) and without AGD (B), while pTau accumulation was highest in ProS in PSP cases with AGD (A) and without AGD (B), but this difference was not significant in the PSP cases with AGD. There was also no predominance of CA2 in either group. In cases with LATE-NC and without neuronal loss, pTDP-43 showed a tendency to be more abundant in CA1 and ProS, but this difference was not significant (C). On the other hand, no predominance of CA1 and ProS was observed in cases with LATE-NC with neuronal loss (D). *p<0.05, **p<0.01, ***p<0.001, ****p<0.0001 with Steel multiple comparison test
